# Magnesium Bicarbonate–Walnut Shell Dual-Template Synthesis of Multifunctional Layered Porous Carbon for Enhanced Adsorption of Aqueous Chlorinated Organic Compounds

**DOI:** 10.3390/ijms252111761

**Published:** 2024-11-01

**Authors:** Juanxue Kang, Xiaoli Bai, Junyang Leng, Yaxuan Lou, Daomei Chen, Liang Jiang, Jiaqiang Wang

**Affiliations:** 1 School of Chemical Sciences & Technology, School of Materials and Energy, Institute of Frontier Technologies in Water Treatment, National Center for International Research on Photoelectric and Energy Materials, Yunnan University, Kunming 650091, China; kangjuanxue@163.com (J.K.); 18213412529@163.com (X.B.); ljy262@163.com (J.L.); luoyaxuan@stu.ynu.edu.cn (Y.L.); dmchen@ynu.edu.cn (D.C.); jiangliang@ynu.edu.cn (L.J.); 2 Institute of International Rivers and Eco-Security, Yunnan University, Kunming 650091, China; 3 School of Engineering, Yunnan University, Kunming 650091, China

**Keywords:** magnesium bicarbonate, multifunctional walnut shell porous carbon, new cyclic chlorine disinfection by-products, adsorption, coke wastewater

## Abstract

Chloride ions readily react with organic matter and other ions, resulting in the formation of disinfection by-products (DBPs) that exhibit heightened levels of toxicity, carcinogenicity, and mutagenicity. This study creatively employed waste walnut shells as self-templates and low-cost magnesium bicarbonate as a rigid template to successfully synthesize multifunctional porous carbon derived from walnut shells. Employing a series of characterization techniques, it was ascertained that the porous carbon material (WSC/Mg) synthesized via the dual-template method exhibited a distinct layered microscopic surface structure, with a predominance of C and O elements on the surface. The material displayed a high degree of graphitization, significant specific surface area, and abundant oxygen-containing surface functional groups. The incorporation of magnesium bicarbonate as a hard template improved the structure of the walnut shell porous carbon, resulting in a significant enhancement in mass transfer efficiency for the target product on the adsorbent and a substantial improvement in removal efficiency. In comparison with walnut shell-derived carbon using only self-templating, WSC/Mg exhibited a 17.26-fold increase in adsorption capacity for 2,4-dichlorophenol. Furthermore, even after four adsorption–desorption cycles, WSC/Mg-12 maintained an adsorption efficiency above 90%. It is remarkable that WSC/Mg-12 demonstrated exceptional resistance to interference from natural organic matter and pH variations. Moreover, the adsorbed saturated WSC/Mg-12 effectively treated real coke wastewater, resulting in an 80% color removal rate, 20% COD removal rate, and 15% ammonia nitrogen removal rate. In conclusion, this study presents an innovative approach for cost-effective and versatile porous carbon materials with extensive applications in water environment purification and biomass utilization.

## 1. Introduction

Since the global outbreak of the novel coronavirus, daily disinfection and sterilization have become standard practice. Chloride ions are widely employed as the preferred option for this purpose [1,2,3]. However, chloride ions readily react with organic matter, pollutants, and other ions to generate disinfection by-products (DBPs) [4,5,6]. Numerous studies have reported on the toxicity, carcinogenicity, and mutagenicity associated with most DBPs [7]. Over 800 species of DBPs have been identified in the water system. Among them, a new cyclic chlorine disinfection by-product, chlorophenol DBP, stands out due to its stable C−Cl bond and aromatic benzene ring structure; it has been classified as a priority pollutant by several countries, including China [8,9]. Technologies based on biology [10,11], chemistry [12,13,14] and physics [15,16] are utilized for treating chlorophenol DBPs in water. Among these technologies, adsorption technology is considered the optimal choice due to its operational simplicity and cost-effectiveness [17]. Hierarchical porous carbon, which is economical and environmentally friendly, is widely recognized as a highly promising adsorbent material [18].

Three effective approaches for hierarchical porous carbon materials with uniform pore structures include the employment of hard templates, soft templates, and self-templates [19,20]. In terms of hard templates, magnesium oxide (MgO) has garnered significant attention and application due to its distinct two-dimensional sheet or three-dimensional cluster crystal structure [21,22]. By using MgO as a template, the manipulation of mesoporous pores and the formation of a three-dimensional honeycomb layered structure in porous carbon can be regulated, resulting in a significant increase in specific surface area [23,24]. Additionally, the precursors of magnesium oxide, including magnesium acetate, magnesium chloride, magnesium citrate, and magnesium carbonate, have also garnered significant interest among researchers [25,26]. Based on the above, our research group has investigated a novel precursor of magnesium oxide—magnesium bicarbonate. The decomposition of magnesium bicarbonate not only leads to the formation of magnesium oxide but also releases carbon dioxide and water vapor, which are commonly employed gases in physical activation methods [27,28].

Biomass is the most commonly utilized material in self-templating methods, and it is cost-effective, readily available, and has a distinctive microstructure [15,18,29]. However, the limited content of self-templates significantly constrains the enhancement of pore structure. Therefore, synergistically combining both template methods can fully exploit their respective strengths to maximize the abundance and performance of pore structures. The synthesis of high-performance porous carbon adsorbents was achieved through the implementation of a novel dual-template strategy utilizing molten salts (LiBr/KBr) and hydrogen-bonded triazine molecules [30]. The introduction of Mg_5_(OH)_2_(CO_3_)_4_ and ZnCl_2_ into various common biomass precursors as hard templates facilitated the formation of mesopores and macropores in their resulting hierarchically porous carbon architectures [31]. Furthermore, a glucose–zinc and urea dual-template strategy was employed to fabricate porous carbon with a large specific surface area and highly concentrated nitrogen sites [32]. These studies provide confirmation that the dual-template approach represents a viable new strategy.

Currently, a wide range of carbon-based adsorbents, including walnut shells, are extensively employed in water purification technology, and research on water treatment is being conducted with a strong emphasis on sustainability [33,34,35]. The authors’ home region, Yunnan Province, is renowned for having the largest walnut plantation area in China, resulting in a substantial annual production of waste from walnut shells. Its accumulation and subsequent landfilling not only contribute to environmental pollution but also represent a wasteful disposal of valuable biomass resources [36]. Leveraging the regional advantage of the researchers, this study devised a method that combines the benefits of hard templates and self-templates. This involves using waste walnut shells and inexpensive magnesium bicarbonate as self-templates and hard templates to pre-fabricate porous carbon with rich pore structure, high specific surface area, and numerous contact points to significantly enhance material adsorption performance.

This study manipulated the mass ratio of walnut shells and magnesium bicarbonate to synthesize a series of walnut shell porous carbon materials (WSC/Mg) via high-temperature one-step carbonization and thermal decomposition. The investigation probed into the influence of different mass ratios of walnut shell porous carbon materials (WSC/Mg) on the adsorption performance of chlorophenols, and further delved into the adsorption kinetics, adsorption isotherm, and adsorption mechanism of WSC/Mg-12 for chlorophenols. Additionally, this study investigated the interference resistance of WSC/Mg-12 in various aqueous environments and at different pH levels, while also evaluating the material’s reproducibility. Meanwhile, the adsorption treatment of coal tar wastewater was conducted using saturated materials, and the levels of chemical oxygen demand (COD)and ammonia nitrogen were measured before and after adsorption. It is worth noting that limited research has been conducted on the adsorption performance of chlorophenols by waste walnut shell multi-functional layered carbon materials and its practical application in wastewater treatment. This mutually beneficial strategy of utilizing waste for waste treatment warrants further exploration.

## 2. Results and Discussion

### 2.1. Characterization

**Figure 1:**
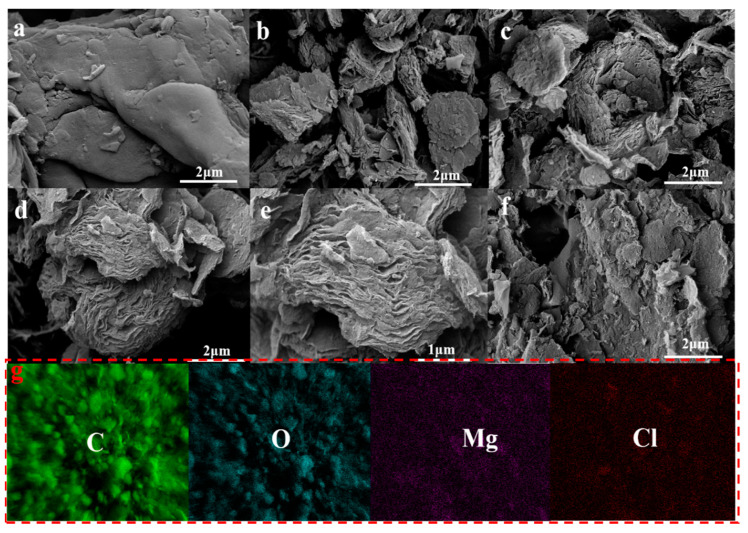
SEM images of (**a**) WSC; (**b**) WSC/Mg-21; (**c**) WSC/Mg-11; (**d**,**e**) WSC/Mg-12; and (**f**) WSC/Mg-13. (**g**) Element mapping of WSC/Mg-12.

Magnesium bicarbonate was utilized as a hard template in the incorporation of waste walnut shells, leading to the synthesis of dual-template porous carbon at mass ratios of m(walnut shell)/m(magnesium bicarbonate) of 1:2, 1:1, 1:2, and 1:3. Subsequent characterization revealed the influence of different mass ratios on material morphology, pore structure, surface functional groups, and element composition. Scanning electron microscopy was utilized for the analysis of the microscopic characteristics of the materials, and the findings are presented in Figure 1. The surface morphology of WSC prepared using the self-template method exhibits minimal folds without any discernible pores or layered structures (Figure 1a), which is consistent with previous research outcomes [37]. In comparison to Figure 1a, Figure 1b–e illustrate varying degrees of porous structure on the material surfaces. Notably, when magnesium bicarbonate is introduced as a hard template, WSC/Mg-21 displays small-sized loose layered structures on its micro-surface. Distinct lamellar and layered structures are observed for WSC/Mg-11 in Figure 1c. At a magnification level of 2 μm in Figure 1d, a rose-like layered structure can be observed, while further enlargement to 1 μm (Figure 1e) reveals an onion-like laminar flow structure for WSC/Mg-12. With increasing amounts of magnesium bicarbonate added, the lamellar structure disappears in WSC/Mg-13, leaving behind only a loosely packed flocculent structure (Figure 1f). The complete removal of the template (WSC/Mg-12) was confirmed via element mapping and EDS analysis, and the obtained results are presented in Figure 1g and Figure S1. The predominant elements detected in WSC/Mg-12 were carbon and oxygen (88.23% and 10.30%), while magnesium and chlorine (0.10% and 0.07%) contents were found to be negligible. These EDS parameters are elaborated on in Table S1. The microstructure of WSC/Mg, prepared using the dual-template method, has been modified to optimize the exposure of adsorption sites and enhance adsorption efficiency [37]. The current approach to achieving a layered carbon structure in biomass materials typically involves compositing them with bimetallic hydroxide [38]. In contrast, utilizing magnesium bicarbonate as a hard template combined with walnut shells offers a more environmentally friendly and cost-effective method of preparation.

**Figure 2:**
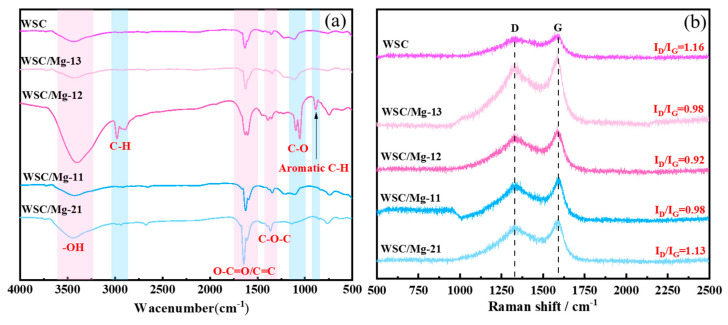
(**a**) FT-IR and (**b**) Raman spectra of WSC, WSC/Mg-21, WSC/Mg-11, WSC/Mg-12, and WSC/Mg-13.

FT-IR spectra were used to analyze the surface functional groups in WSC and WSC/Mg, as shown in Figure 2a. All spectra exhibit a broad and intense absorption band centered at 3440 cm^−1^, corresponding to the stretching vibration peak of intermolecular hydrogen bonding −OH. The vibration observed near 1639 cm^−1^ is attributed to the stretching vibration of O−C=O or C=C in the lignin aromatic ring, while the vibration near 1360 cm^−1^ is attributed to the C−O−C peak of phenolic hydroxyl group. Notably, peaks at 2979 cm^−1^ and 2892 cm^−1^ are present in WSC/Mg-12, indicating asymmetric and symmetric stretching vibrations of sp^3^-hybridized hybridized C−H groups, respectively. Furthermore, the peaks at 1097 cm^−1^ and 1054 cm^−1^ correspond to asymmetric and symmetric stretching vibrations of both aromatic and aliphatic ether C−O groups. The distinct peak at 890 cm^−1^ can be attributed to the presence of an aromatic ring structure [39,40,41]. The emergence of these new peaks in WSC/Mg-12 when the mass ratio of walnut shell to magnesium bicarbonate is 1:2 indicates that, at this ratio, the carbon dioxide and water vapor released by magnesium bicarbonate react with the surface of walnut shell carbon material, leading to the formation of novel functional groups. Considering the presence of diverse surface functional groups on these materials, such as −OH, C−H, C=C/O−C=O, C−O−C, and C−O, it can be inferred that these functional groups may play a crucial role in the adsorption of DBP.

The Raman spectra of WSC and WSC/Mg are illustrated in Figure 2b. All samples exhibit two distinct broad bands: the D band (1320 cm^−1^), which is associated with sp^3^ carbon atom disorder, and the G band (1598 cm^−1^), which is related to the sp^2^ hybridization structure of graphite carbon. The intensity ratio (I_D_/I_G_) of the D and G bands serves as an indicator for evaluating the degree of disorder in the carbon material, with a lower value indicating higher graphitization [42]. The I_D_/I_G_ values for the WSC, WSC/Mg-21, WSC/Mg-11, WSC/Mg-12, and WSC/Mg-13 samples are 1.1552, 1.1313, 0.9864, 0.9190, and 0.9834, respectively. The I_D_/I_G_ ratio of the sample initially decreases and subsequently increases with increasing magnesium bicarbonate content in the hard template, indicating that the thermal decomposition of magnesium bicarbonate into magnesium oxide enhanced the degree of graphitization of the material [43]. It is noteworthy that the I_D_/I_G_ ratio of the WSC/Mg-12 sample is the smallest, suggesting its highest degree of graphitization. In DBP adsorption experiments, a highly graphitized material facilitates electron transfer to the benzene ring of DBP, leading to π-π stacking [44], thereby improving its adsorption performance.

**Figure 3:**
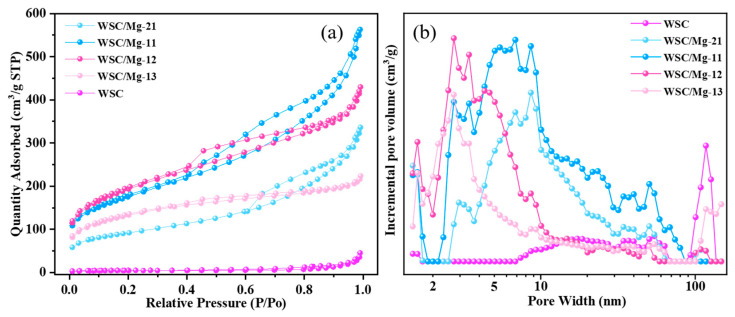
(**a**) N_2_ adsorption–desorption curves; (**b**) pore size distribution of WSC, WSC/Mg-21, WSC/Mg-11, WSC/Mg-12, and WSC/Mg-13.

**Figure 4:**
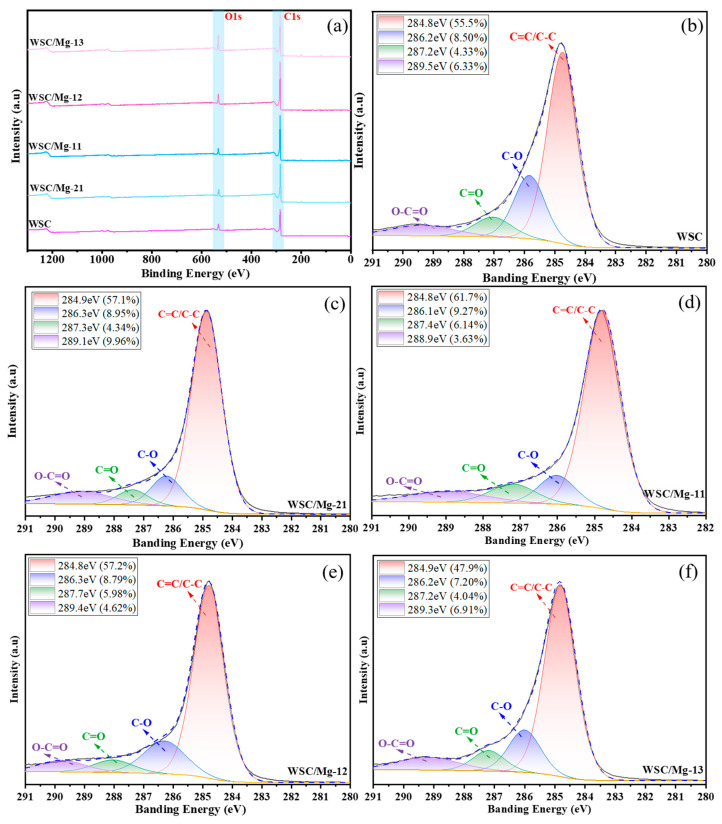
(**a**) XPS; (**b**) C1s of WSC; (**c**) C1s of WSC/Mg-21; (**d**) C1s of WSC/Mg-11; (**e**) C1s of WSC/Mg-12; (**f**) C1s of WSC/Mg-13.

When investigating the adsorption capacity of an adsorbent, it is imperative to consider its specific surface area and pore size, as these are pivotal factors that significantly influence its adsorption performance. The N_2_ adsorption–desorption isotherm for both WSC and WSC/Mg materials is depicted in Figure 3a. According to the IUPAC classification [45], both WSC and WSC/Mg materials display I/IV isotherm adsorption–desorption curves. The adsorption isotherm of WSC/Mg materials rises sharply when P/P_0_ < 0.1, indicating that the sample contains a great quantity of micropores. When the relative pressure of WSC/Mg materials P/P_0_ > 0.2, the adsorption–desorption isotherm has an obvious hysteresis loop shape, and capillary condensation occurs in this region, indicating that the sample has rich mesopores [45]. The pore size distribution of WSC and WSC/Mg materials is shown in Figure 4b. Compared with WSC, WSC/Mg materials exhibit an obvious hierarchical pore structure, with 1.4–2 nm nanopores, containing 2–50 nm mesopores at the main distribution and a limited quantity of macroporous structures. Incorporating magnesium bicarbonate as a hard template during preparation noticeably enhances the abundance of mesoporous pores in the resulting WSC/Mg porous carbon material, which is further elaborated in Table 1. The addition of a magnesium bicarbonate template significantly enhanced the specific surface area of the adsorbed material in comparison to WSC. Specifically, the specific surface areas of WSC/Mg-21, WSC/Mg-11, WSC/Mg-12, and WSC/Mg-13 increased by 20-fold, 39-fold, 44-fold, and 30-fold, respectively. The significant increase in the specific surface area of the WSC/Mg material can be attributed to the thermal decomposition process of magnesium bicarbonate, which involves the release and activation of water vapor and carbon dioxide, respectively, as well as the removal of the hard template formed by magnesium oxide [27,28]. Nevertheless, compared to WSC/Mg-12, WSC/Mg-13 exhibited a lower specific surface area due to excessive obstruction caused by magnesium bicarbonate impeding the self-decomposition process of walnut shell. Significantly, this discovery aligns with the findings from SEM experiments.

XPS analysis was conducted on the biological carbon materials of WSC, WSC/Mg-21, WSC/Mg-11, WSC/Mg-12, and WSC/Mg-13 to further investigate their surface composition. Figure 4a illustrates the overall XPS spectrum, revealing that the surface of the biological carbon material primarily consists of C and O elements. In comparison with the walnut shell self-template, the incorporation of magnesium bicarbonate template led to a substantial enhancement in the response of the C1s peak in the WSC/Mg series. The four primary peaks in the C1s spectrum can be ascribed to the O−C=O bond, C=O bond, C−O bond, and C=C/C−C bond.

Furthermore, three distinct peaks were discernible in the O1s spectrum of WSC, representing the C=O bond, C−O bond, and O−C=O bond (Figure S2). In contrast, only two discernible peaks representing the C−O bond and O−C=O bond were evident in the O1s spectrum subsequent to introducing magnesium bicarbonate template. These modifications resulted in an alteration in the relative proportion of C1s and O1s, as delineated in Table 2 and Table 3. These findings suggest that the gradual oxidation of WSC/Mg materials occurs through the incorporation of magnesium bicarbonate hard templates, resulting in an increase in carboxyl and hydroxyl functional groups [46,47].

The results obtained from the aforementioned characterization methods demonstrate the successful synthesis of porous carbon material derived from walnut shells using the double template method. The incorporation of the magnesium bicarbonate hard template modifies the microstructure, potentially exposing a greater number of contact sites and augmenting surface functional groups. This consequently leads to enhanced graphitization, expanded specific surface area, and pore structure, thereby establishing a solid foundation for improving the properties of walnut shell-based porous carbon.

### 2.2. Effect of Different Materials on Adsorption Capacity

**Figure 5:**
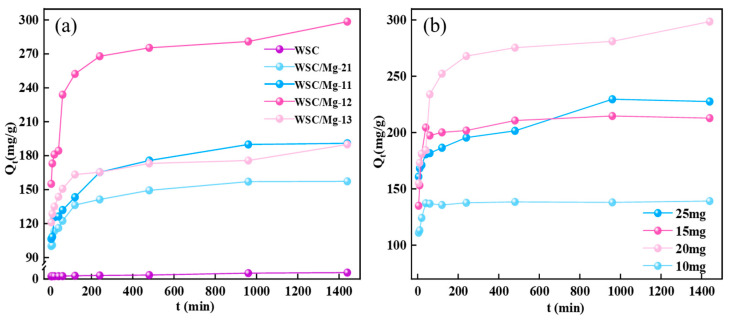
(**a**) The effect of different materials; (**b**) the effect of dosage in 2,4-DCP on WSC/Mg-12.

Figure 5a clearly illustrates the adsorption behavior of walnut shell self-template carbon material (WSC) and dual-template layered carbon materials of magnesium hydrogen carbonate (WSC/Mg-21, WSC/Mg-11, WSC/Mg-12, and WSC/Mg-13) with respect to the novel chlorine-based disinfection by-product (2,4-DCP) as a function of time. The amount of adsorbed 2,4-DCP increases with time. The maximum adsorption capacities of WSC, WSC/Mg-21, WSC/Mg-11, WSC/Mg-12, and WSC/Mg-13 for 2,4-dichlorophenol are 17.26 mg/g, 157.34 mg/g, 190.97 mg/g, 298.56 mg/g, and 189.97 mg/g, respectively; these values are 9.12-fold, 11.06-fold, 17.3-fold, and 11.01-fold higher than those for WSC. The incorporation of magnesium bicarbonate as a hard template significantly enhances the specific adsorption capacity of porous carbon derived from walnut shell for 2,4-DCP. This confirms that the modification in microstructure of WSC/Mg series materials effectively exposes more active sites and increases surface functional groups to facilitate multiple binding mechanisms during the adsorption process with a high degree of graphitization. Electrons originating from WSC/Mg series materials migrate towards the benzene ring of 2,4-DCP, thereby promoting π-π stacking interactions. Simultaneously, the substantial augmentation in specific surface area and abundant pore structure also provides additional pathways for efficient transport and uptake of 2,4-DCP.

In the adsorption experiment, the utilization of adsorbent quantity is also a critical factor for evaluating the adsorption performance. Consequently, subsequent experiments were conducted to further investigate based on the optimal WSC/Mg-12. As depicted in Figure 5b, an increase in 2,4-DCP adsorption by the adsorbent was observed as the amount of added adsorbent increased from 10 mg to 25 mg. Notably, at an addition of 20 mg, the adsorption system exhibited its highest capacity at 295 mg/g; however, this capacity remained unchanged when the addition was raised to 25 mg. Therefore, it was determined that the optimal amount of added adsorbent should be 20 mg. When there are more novel chlorine-based disinfection by-products in solution than active sites on the surface of the adsorbent, there will be a decrease in adsorption capacity below this threshold point. Conversely, exceeding a dose of 25 mg will lead to excessive active sites and result in wasted capacity and reduced equilibrium absorption.

### 2.3. Adsorption Experiment

#### 2.3.1. Adsorption Kinetics

**Figure 6:**
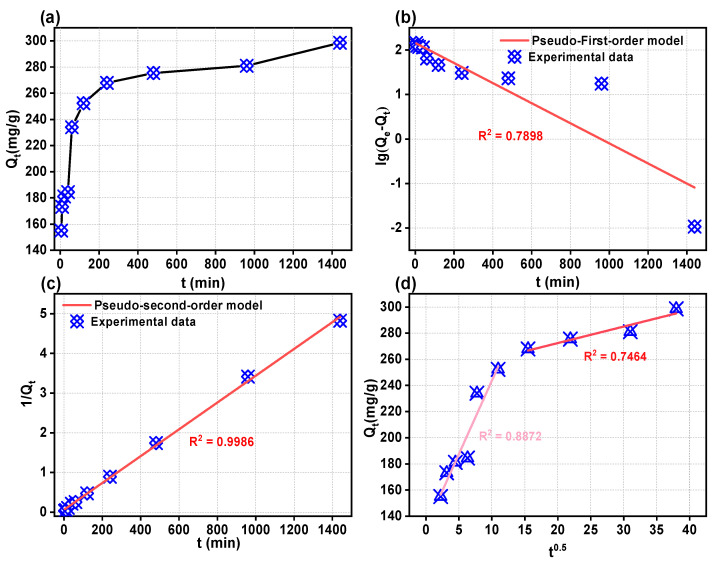
(**a**) The relationship between the duration of adsorption and the amount of adsorption; (**b**) pseudo-first order model; (**c**) pseudo-second order model; (**d**) intra-particle diffusion model of WSC/Mg-12.

This experiment extensively investigated the kinetics of 2,4-DCP adsorption at an ambient temperature of 25 °C and a concentration of 200 mg/L. The findings presented in Figure 6a illustrate the correlation between the quantity of adhered adsorbent material and different durations of adsorption. By applying a kinetic model to analyze our experimental data, valuable insights into the underlying absorption mechanism can be obtained to determine whether it involves physisorption or chemisorption processes. To gain a comprehensive understanding of this mechanism’s dynamics, we calculated both pseudo-first-order and pseudo-second-order rate constants for 2,4-DCP.

The linear forms of these models are as follows [48]:

The pseudo-first-order kinetic model, which is the earliest known model, is expressed as

(1) $$\ln\left(\mathrm{Qe}-\mathrm{Qt}\right)=\mathrm{lnQe}-k_{1}t$$

The pseudo-second order kinetic model is shown as follows:

(2) $$\frac{1}{\mathrm{Qt}}=\frac{1}{k_{2}\mathrm{Qe}}+\frac{1}{\mathrm{Qe}}t$$

In the model, Qt (mg/g) and Qe (mg/g) are the adsorption amounts at t (min) and equilibrium, respectively; k_1_ (min^−1^) and k_2_ (g·mg^−1^·min^−1^) are the quasi-primary and quasi-secondary rate constants, respectively.

The pseudo-first-order kinetic model and pseudo-second-order kinetic model are shown in Figure 6b,c. The pseudo-second-order kinetic model provides a better fit for describing the adsorption behavior between WSC/Mg-12 and 2,4-DCP molecules compared to the pseudo-first-order kinetic model; this conclusion is supported by slightly smaller differences observed between Qe(cal) and Qe. The R^2^ value of the pseudo-second-order kinetic model for the 2,4-DCP molecule (0.9986) exceeds that of the pseudo-first-order kinetic model. Furthermore, the Qe(cal) value obtained from the pseudo-second-order kinetic model (296.7 mg/g) closely matches the experimental Qe(exp) value (298.5 mg/g). These findings validate the conclusion that the pseudo-second-order kinetic model is most suitable for fitting the experimental kinetics data of WSC/Mg-12. Additionally, all adsorption processes of WSC/Mg-12 on 2,4-DCP molecules can be attributed to chemisorption.

To gain a deeper understanding of the diffusion mechanism, the kinetic results were evaluated using an intraparticle diffusion model, as demonstrated in the following equation:

(3) $$\mathrm{Qt}=k_{i}t_{0.5}+C$$

The intra-particle diffusion rate constant, ki (mg^−1^·g·min^−1/2^), and the constant about the boundary layer, C (mg/g), are introduced. When C = 0, the adsorption process is intraparticle diffusion.

A linear plot of Qt fitted to t_0.5_ is presented in Figure 6d. It clearly illustrates that the adsorption process follows a two-stage pattern, characterized by rapid adsorption for approximately 2 h followed by a slower adsorption phase that takes up to 24 h to reach equilibrium. During the initial stage, the WSC/Mg-12 surface exhibits a higher number of active sites, resulting in an accelerated rate of adsorption. The initial phase is likely attributed to external mass transfer, boundary layer diffusion, or membrane diffusion where 2,4-DCP molecules permeate through the membrane to reach WSC/Mg-12. The subsequent linear region represents intraparticle or pore diffusion where 2,4-DCP molecules penetrate into the pores of the adsorbent material. Moreover, the fact that there is a non-zero intercept in the linear regression analysis between Qt and t_0.5_ suggests that a combination of extra- and intra-particle diffusion mechanisms may be involved in this adsorption process. It is important to note that this observation is objective and devoid of any subjective evaluations. The decrease in the concentration of 2,4-DCP molecules in solution during the later stages can be attributed to their gradual occupation of active sites on the adsorbent surface. Consequently, as the possibility of encountering active sites decreases, there is a deceleration in the rate of adsorption until equilibrium is attained. The kinetic adsorption parameters on WSC/Mg-12 are shown in Table 4.

#### 2.3.2. Adsorption Isotherms

To understand the feasibility, performance, capacity, and mechanism of an adsorption system, it is necessary to fit adsorption isotherms [49,50]. To achieve this, we conducted adsorption fitting experiments using four different models: Langmuir, Freundlich, Temkin, and Sips [51,52]. These experiments were conducted for a concentration range of 50 mg/L–400 mg/L of 2,4-DCP.

The Langmuir isotherm model, expressed in its nonlinear form, is as follows:

(4) $$\mathrm{Qe}=\frac{Q_{\max}C_{e}k_{L}}{1+C_{e}k_{L}}$$

In the model, Ce is the equilibrium concentration of 2,4-DCP after adsorption (mg/L), Qe and Q_max_ are the adsorption capacity at equilibrium and the calculated maximum adsorption capacity (mg/L), and k_L_ (L/mg) is the Langmuir constant.

The nonlinear format of the Freundlich model is represented by the following equation:

(5) $$\mathrm{Qe}=K_{F}{\mathrm{Ce}}^{1/n}$$

The equilibrium concentration (Ce, mg/L) is determined by the adsorption capacity (K_F_, mg^(1−1/n)^ L^1/n^g^−1^), and the adsorption strength is represented by 1/n.

The R_L_ parameter in the Langmuir, Temkin, and Sips temperature adsorption model equations are shown in the Supplementary Material, Text S1.

**Figure 7:**
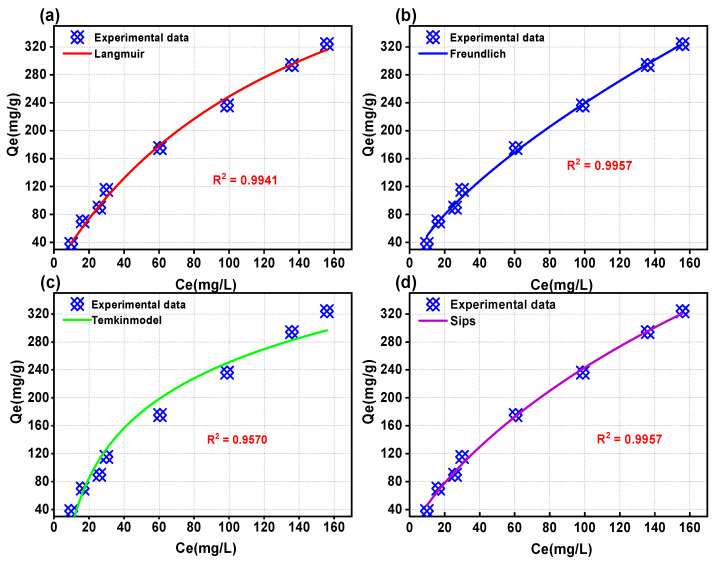
Adsorption isothermal models of WSC/Mg-12: (**a**) Langmuir; (**b**) Freundlich; (**c**) Temkin; (**d**) Sips.

The Langmuir model assumes a homogeneous surface for adsorption without any interaction between the adsorbed molecules [53]. On the other hand, the Freundlich isotherm model describes empirical equations for heterogeneous surfaces and considers an increasing interaction strength between the adsorbent and adsorbate molecules with higher degree of site occupation [50]. The Sips model serves as a suitable adsorption isotherm model for elucidating complex adsorption systems, effectively bridging the gap between the Langmuir and Freundlich models. The isothermal adsorption models of Langmuir, Freundlich, Temkin, and Sips are shown in Figure 7a–d. The parameter values of the four adsorption isotherms are presented in Table 5. The correlation coefficients (R^2^ values) for the Langmuir, Freundlich, Temkin, and Sips adsorption isothermal models were determined to be 0.9941, 0.9957, 0.9570, and 0.9957, respectively. With the exception of the Temkin model, all other models exhibited correlation coefficients exceeding 0.99. It can be observed from the tabulated data that the Langmuir constant (K_L_) signifies a significant strength of interaction between the adsorbent and adsorbate, with a noteworthy value of K_L_ = 1.225 mg/L. The maximum adsorption capacity of WSC/Mg-12 was found to be 610.6 mg/g, while the RL factor ranged between 0.0021 and 0.0161, indicating favorable adsorption (i.e., within the range of 0 < R_L_ < 1). These results demonstrate an excellent adsorbability of WSC/Mg-12 towards the compound 2,4-DPC. The R^2^ values for the Freundlich and Sips adsorption isotherm models consistently surpass those obtained from Langmuir model, suggesting their suitability for describing the process of 2,4-DPC adsorption on WSC/Mg-12 surface which further indicates a heterogeneous multimolecular layer-based mechanism for this particular system’s adsorption process. In the Freundlich model, n represents the adsorption capacity and strength index of the corresponding system, with 1/n = 0.6835 in the Freundlich isotherm model indicating a high affinity between chlorophenol and WSC/Mg-12 [15]. Comparing the Langmuir model fitting data for 2,4-DCP (R^2^ = 0.9949) with the Freundlich isotherm fitting data (R^2^ = 0.9957), the multi-molecular layer adsorption of chlorophenol 2,4-DCP by physical and chemical adsorption processes can be observed. 

#### 2.3.3. Adsorption Mechanism Analysis

**Figure 8:**
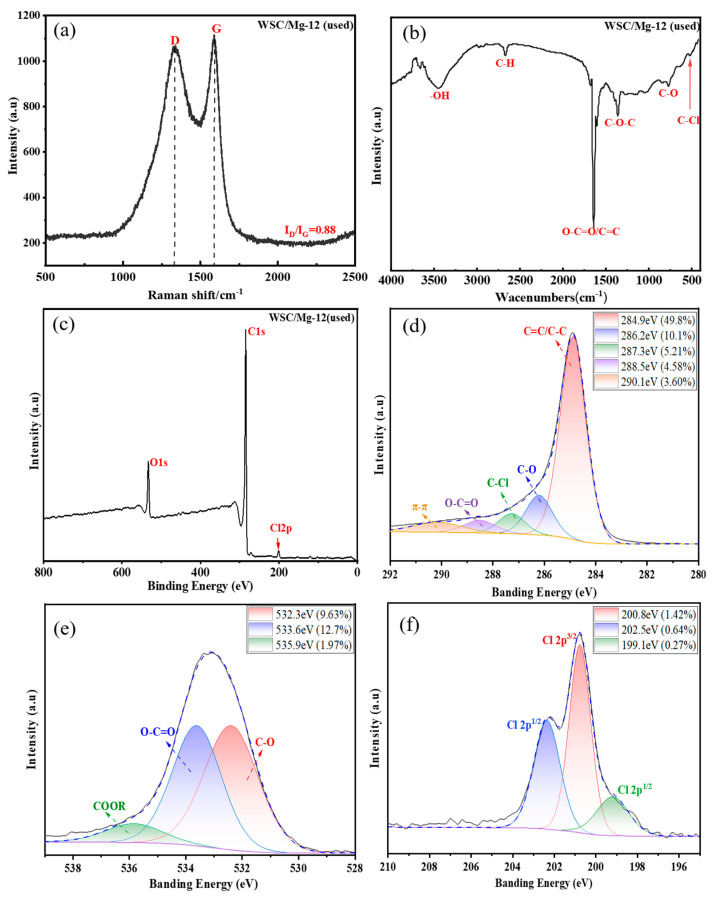
(**a**) Raman spectra, (**b**) FT-IR spectra, and (**c**) XPS of WSC-12 (used); (**d**) C1s, (**e**) O1s, and (**f**) Cl2p of WSC-12 (used).

As previously mentioned, intermittent adsorption experiments have provided evidence for the presence of electrostatic interactions and physical or chemical effects between WSC/Mg-12 and DBP. To further validate these speculations, Raman spectroscopy, FT-IR spectroscopy, XPS, and SEM were employed to characterize the adsorbed WSC/Mg-12. Figure 8a illustrates the adsorption interaction of WSC/Mg-12 on 2,4-DCP through Raman spectroscopy. After chlorophenol adsorption, a decrease in the I_D_/I_G_ value from 0.92 to 0.88 was observed, indicating an increase in the sp^2^ structure region during the graphitization of the material and providing additional support for the existence of π–π bonds [54,55]. The infrared spectrum analysis in Figure 8b reveals a vibration peak at 511 cm^−1^, corresponding to C−Cl bond stretching [41]. The XPS spectrum in Figure 8c exhibits a new Cl 2p peak at 201.1 eV; narrow-spectrum high-resolution scanning was performed for more detailed analysis. Notably, Figure 8d presents a fine C1s spectrum with a new C−Cl peak at 287.3 eV and a π-π peak at 290.1 eV, providing sufficient evidence for the successful adsorption of aqueous chlorinated organic compounds by WSC/Mg-12. Additionally, groups of COOR appear at 535.9 eV in the O1s spectrum shown in Figure 8e, while orbital configurations capable of receiving electrons emerge in the Cl2p spectrum depicted in Figure 8f [15].

**Figure 9:**
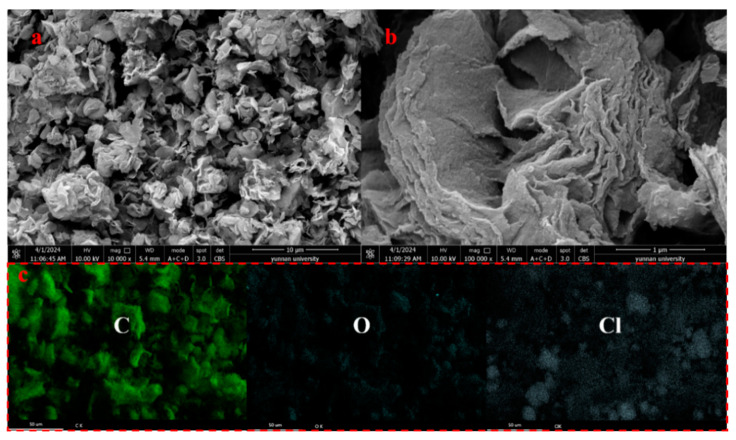
(**a**) 10,000× and (**b**) 100,000× SEM images of WSC-12 (used); (**c**) mapping of WSC-12 (used).

**Figure 10:**
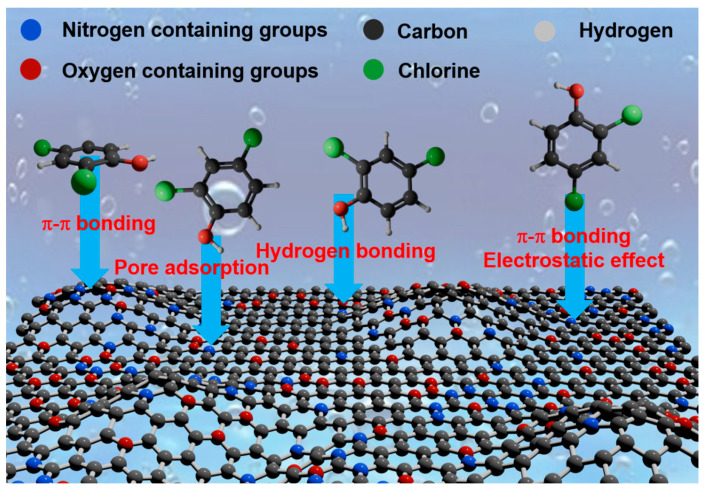
Schematic illustration of possible adsorption mechanism.

Figure 9 depicts the SEM images and mapping results, indicating no significant alteration in the morphology of the adsorbent before and after adsorption. Additionally, the mapping analysis demonstrates a uniform chlorine adsorption onto porous carbon WSC-12. In the EDS analysis of WSC/Mg-12 (used) (shown in Figure S3), the predominant elements were carbon, oxygen and chlorine (86.88%, 4.79%, and 7.55%). These findings are elaborated on in Table S2. Based on the adsorption kinetics, adsorption isotherm, and characterization of materials before and after adsorption, the main mechanism of 2,4-DCP adsorption primarily relies on pore adsorption, π-π bonding, and electrostatic adsorption. All forms of DBP(2,4-DCP) adsorption by WSC/Mg-12 are displayed in Figure 10.

### 2.4. Anti-Interference Performance

The pH of a solution is a very critical factor affecting its adsorption behavior. Solution pH not only affects the electrical properties of functional groups on the adsorbent surface, but also plays a decisive role in the ionization state of the adsorbate. Consequently, the pH of the solution can serve as an indicator of the adsorbent of resilience to interference. The influence of initial solution pH on the adsorption behavior of 2,4-DCP was investigated under conditions with an initial concentration of 50 mg/L and a range of initial pH values from 3 to 9. Figure S4a illustrates the adsorption and removal performance of WSC/Mg-12 for 2,4-DCP at different pH levels. The adsorption capacity of WSC/Mg-12 for 2,4-DCP gradually increased as the pH increased until it reached its maximum at pH = 9. Overall, both the removal rate and adsorption capacity exhibited consistent changes with increasing pH; however, regardless of variations in pH levels, WSC/Mg-12 achieved a minimum removal rate above 89%, with a peak removal rate reaching up to 95%.

A zeta potential analysis of WSC/Mg-12 was conducted across a pH range of 2 to 10 in order to gain deeper insights into the pH-dependent performance of this material, as shown in Figure S4b. The pH pzc for WSC/Mg-12 was determined to fall within the range of 3 and 4, with a linear fitting result yielding a value of pH pzc = 3.61. When the initial solution pH is below the pH pzc, adsorption primarily occurs through ion exchange mechanisms. As the solution pH surpasses the threshold value of 3.61, the surface charge on WSC/Mg-12 becomes positively charged, thereby enhancing electrostatic attraction during the adsorption process. At this stage, adsorption is driven by van der Waals forces, intramolecular diffusion, electrostatic attraction, and ion exchange mechanisms. However, when the solution pH > 6, noticeable electrostatic attraction arises between positively charged WSC/Mg-12 and negatively charged molecules of 2,4-DCP, promoting their adsorption onto WSC/Mg-12 [56].

**Figure 11:**
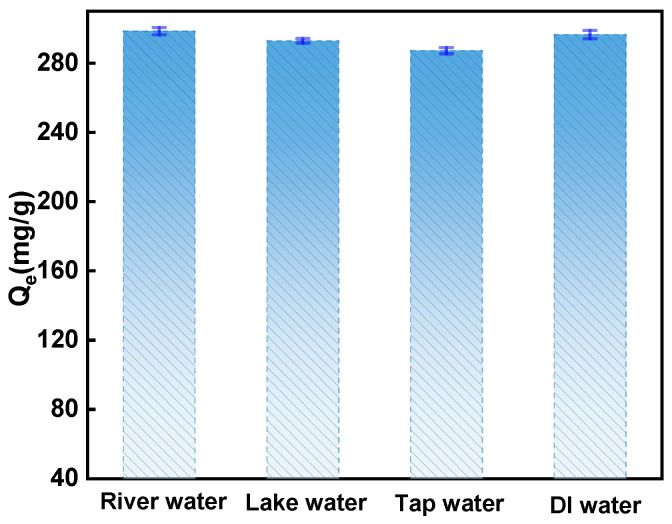
Adsorption of 2,4-DCP in different water samples.

The adsorbent’s anti-interference capability extends beyond pH-induced performance changes, as it demonstrates effective adsorption in complex real water environments. To accurately simulate practical usage scenarios, four distinct water samples were employed in the experiment. These samples were utilized to prepare a novel circulating chlorine disinfection by-product (2,4-DCP) with a concentration of 200 mg/L. Figure 11 presents the results at adsorption equilibrium. It is evident from the figure that the influence of these four complex real water samples on WSC/Mg-12 adsorption efficiency is negligible, indicating the material’s stability and robust anti-interference properties.

### 2.5. Recyclability and Applicability Study

**Figure 12:**
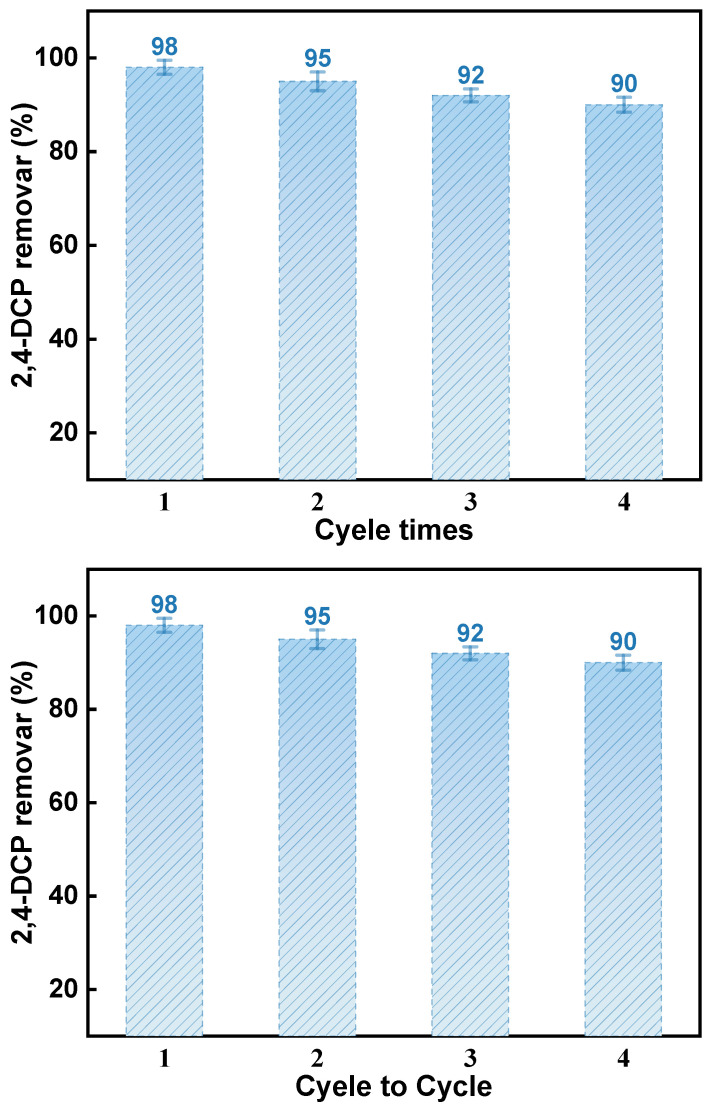
Recyclability of WSC/Mg-12.

The recyclability of materials is a crucial factor in determining their reusability and economic viability. Following adsorption experiments, desorption and regeneration studies were conducted to assess the material’s recyclability and stability for practical applications, as shown in Figure 12. After adsorbing 2,4-DCP, waste samples were collected and dried at 60 °C for 4 h. The spent sample was then calcined at 450 °C to decompose 2,4-DCP [57]. The figure shows a decrease in the adsorption capacity of the material for 2,4-DCP with each recovery test. This decrease suggests that some of the adsorption sites are occupied by residual 2,4-DCP molecules, and the adsorbent becomes inhomogeneous after each experimental operation. However, after four recycle runs, the sorbent’s adsorption capacity remained above 90% of the original value, demonstrating the material’s good recoverability.

As illustrated in Table 6, WSC-12 exhibits minimal variations in the maximum adsorption capacity of chlorophenol 2,4-DCP within a pH range of 3–9 when compared to other chlorophenol adsorbents. Despite possessing a lower specific surface area than NC-1000@PAA, WSC-12 surpasses its maximum adsorption capacity. This implies that while material-specific factors such as specific surface area and pore structure can influence performance, they do not solely determine the adsorption capabilities. With its wide pH range, anti-interference properties, and excellent stability, WSC-12 showcases promising potential for various novel cyclic DBPs’ adsorption treatments.

### 2.6. Multifunctional Performance

The indicators used to evaluate an adsorption material include not only its stability, high efficiency, and anti-interference but also its multi-functionality in absorbing diversified target pollutants, which is the focus of this experimental research. The saturated materials that absorbed 2,4-DCP were recovered and then dried in an oven at 60 °C for 12 h. The experimental water sample used to investigate the multifunctional adsorption materials was wastewater produced by Dawei Coking Gas Supply Co., Ltd., in Qujing, China. A porous carbon material made from walnut shells after adsorbing 2,4-DCP was weighed at 20 mg and placed in a 50 mL centrifuge tube with 50 mL coking wastewater added. The entire adsorption process was oscillated at 150 rpm under a constant temperature oscillator for 24 h. To reduce experimental errors, three sets of identical experiments were conducted to obtain average values for evaluating the limitations of material application. The actual composition of coking wastewater is more complex; therefore, COD (chemical oxygen demand), ammonia nitrogen, and color were used as evaluation indicators for the material. The experimental results are shown in Table 7: the COD value decreased from 2260 mg/L to 1819 mg/L, and the ammonia nitrogen value decreased from 447.47 mg/L to 378 mg/L. These findings indicate an effective removal of both organic and inorganic matter by the adsorption materials.

**Figure 13:**
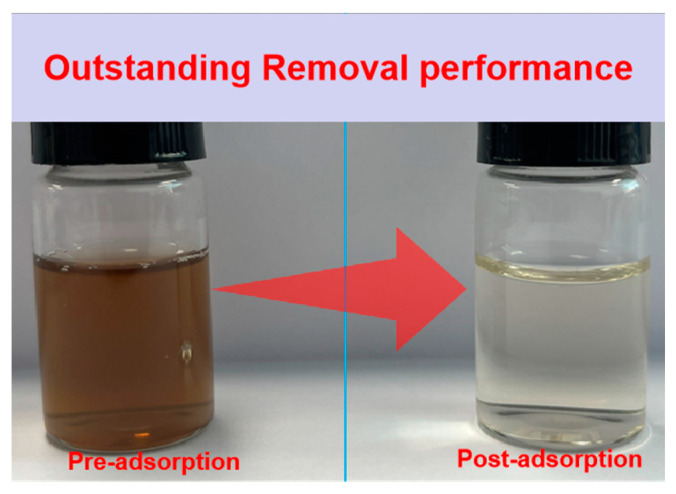
Comparison of coking wastewater pre-adsorption and post-adsorption.

The water sample of coking wastewater before and after adsorption is depicted in Figure 13. It can be observed from the figure that substantial alterations occurred in the water sample, with the post-adsorption sample exhibiting near-clarity. This observation signifies the successful synthesis of multi-functional layered porous carbon, wherein the WSC/Mg material not only demonstrates stability, efficiency, and anti-interference properties but also possesses a higher capacity for adsorbing target pollutants. Moreover, its adsorption characteristics towards coking wastewater are notably superior.

## 3. Materials and Methods

### 3.1. Synthesis of WSC/Mg

Information on the materials used is listed in the Supplementary Materials, Text S1.

**Figure 14:**
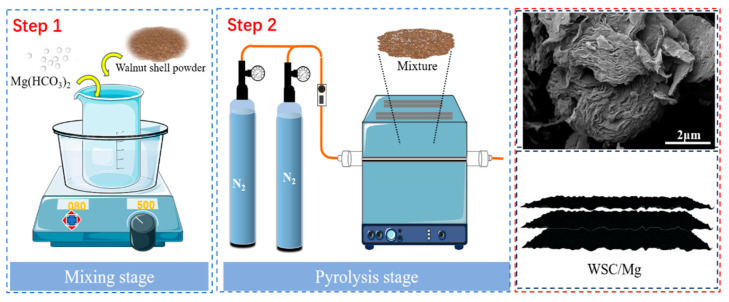
Schematic representation of WSC/Mg preparation procedure.

The walnut shells (WSs) were thoroughly rinsed with distilled water, air dried naturally, pulverized using a flour beater, and subsequently sieved to obtain 200-mesh walnut shell powder for subsequent utilization. A total of 1 g of walnut shell powder was taken and placed in a 50 mL beaker. Then, 10 mL of deionized water and different quantities of magnesium bicarbonate were added. The components were thoroughly mixed and the mixture was heated in an 80 °C water bath until it formed a paste. The mixture was allowed to dry overnight at 100 °C before being transferred to a 50 mL quartz boat. It underwent calcination under an N_2_ atmosphere at 700 °C for 2 h (heating rate of 3 °C/min), followed by cooling to room temperature before being retrieved as a black product. To eliminate any unreacted magnesium bicarbonate, the material was immersed in a solution of HCl with a concentration of 2 mol/L (S/V = 1/20). The stirring process was continued for a duration of two hours, followed by multiple rinses with deionized water until the material achieved a neutral pH. Then, it was dried overnight at 60 °C, ground into a powder, and stored at room temperature for future use. The materials were named according to the different mass ratios of walnut shell powder to magnesium bicarbonate; for example, WSC/Mg-21 stood for a mass ratio of 2:1 (m walnut shell/m magnesium bicarbonate). Samples WSC/Mg-21, WSC/Mg-11, WSC/Mg-12, and WSC/Mg-13 were synthesized. In addition, to facilitate comparison, the same conditions were used to prepare walnut shell biochar without magnesium bicarbonate (WSC). The preparation process of WSC/Mg is shown in Figure 14.

### 3.2. Adsorption Experiments

Batch adsorption experiments were conducted to investigate the removal performance of the new cyclic chlorine disinfection by-product solution, and the corresponding information is listed in Table S3. The experiments were carried out in a 50 mL centrifuge tube at a temperature of 25 °C. The entire adsorption process was fully oscillated at 150 rpm using a constant temperature oscillator for 24 h to evaluate various parameters that affect the removal of the 2,4-DCP solution. The pH of the 2,4-DCP solution was adjusted by titration using a 0.1 mol/L hydrochloric acid/sodium hydroxide solution, and the influence of the adsorbent on pH was investigated. The adsorption kinetics of the 2,4-DCP solution were examined at various time intervals. In order to investigate its adsorption capacity, the adsorption isotherms of a 2,4-DCP solution with an initial concentration ranging from 50 to 400 mg/L were examined. Once equilibrium was reached, the supernatant was filtered using a 0.22 μm filter and its absorbance was measured using a UV Vis spectrophotometer (Shimadzu UV 2600 photometer, China). Information on instrumental parameters is displayed in detail in the Supplementary Material, Text S2. The concentrations of 2,4-DCP were determined using a UV Vis spectrophotometer at λ = 284 nm. To ensure data reproducibility, each experiment was measured in parallel at least three times. 

2,4-DCP was calculated using the following equation:

(6) $$\mathrm{Removal}\%=\frac{\left(C-\mathrm{Ce}\right)}{C}\times 100$$

The instant adsorption amount Qt (mg/L) of 2,4-DCP was evaluated using the following formula:

(7) $$\mathrm{Qt}=\left(C-\mathrm{Ct}\right)\times V/m$$

The equilibrium adsorption amount Qe (mg/L) of 2,4-DCP was evaluated using the following equation:

(8) $$\mathrm{Qe}=\left(C-Ce\right)\times V/m$$

Above, C (mg/L), Ct (mg/L), and Ce (mg/L) are the initial, instant, and equilibrium concentrations of the 2,4-DCP solution, respectively. V (L) is the volume of the solution and m (g) is the weight of the adsorbent.

### 3.3. Detection of Environmental Water Samples

The aquatic environment contains a complex mixture comprising both organic and inorganic substances that have the potential to affect the efficiency with which 2,4-DCP is adsorbed. In order to assess the ability of WSC/Mg-12 to adsorb 2,4-DCP present in real-world water samples accurately, we collected various types of such samples from Yunnan University Chenggong Campus (Kunming, China). No traces of this compound were detected in any of the sampled waters; thus, a solution was prepared using these environmentally sourced waters at an initial concentration level of approximately 200 mg/L. The experiments were conducted by adding 20 mg of the adsorbent to 50 mL of the solutions prepared from different water samples. The pH of the solutions was not adjusted to ensure that a real-world environment was simulated.

## 4. Conclusions

In summary, a multifunctional layered porous carbon adsorbent (WSC/Mg) was successfully synthesized via magnesium bicarbonate dual templates. The resulting WSC/Mg-12 exhibited excellent stability, remarkable adsorption properties, and wide applicability. Notably, WSC/Mg-12 demonstrated significant adsorption performance for 2,4-DCP in various types of environmental water samples. This can be attributed to its uniform surface charge distribution, abundant layered porous structure with surface functional groups, and high degree of graphitization that facilitates strong binding with cyclic chlorine disinfection through electrostatic interaction, π-π interaction, pore adsorption, and hydrogen bonding. Moreover, WSC/Mg-12 displayed exceptional stability and anti-interference ability while effectively removing 2,4-DCP from diverse environmental water samples. Additionally, this material also exhibited superior adsorption characteristics for complex actual coking wastewater by reducing the COD value by 440 mg/g and ammonia nitrogen by 69 mg/g. The proposed WSC/Mg-12 exhibits promising potential as a competitive candidate material for efficient pollutant removal from environmental water samples, due to its facile preparation, inherent stability, eco-friendliness, exceptional adsorption properties, and wide applicability.

## Figures and Tables

**Table 1:** The S_BET_, S_micro_, pore parameters, and BJH average pore diameter for WSC, WSC/ Mg-21, WSC /Mg-11, WSC/Mg-12, and WSC/Mg-13.

Adsorbent.	S_BET_ (m^2^/g)	S_micro_ (m^2^/g)	V_total_ (cm^3^/g)	V_micro_ (cm^3^/g)	BJH Average Pore Diameter (nm)
WSC	16.07	9.241	0.06605	0.004161	9.335
WSC/Mg-21	324.7	78.59	0.2834	0.03477	5.538
WSC/Mg-11	630.2	83.97	0.5150	0.03295	4.901
WSC/Mg-12	696.1	82.64	0.3834	0.03054	3.408
WSC/Mg-13	475.9	81.55	0.2858	0.03250	2.671

**Table 2:** Quantitative analysis of C species in WSC, WSC/Mg-21, WSC/Mg-11, WSC/Mg-12, and WSC/Mg-13 through C1s XPS spectra.

Attribution	Relative Content (%)				
	WSC	WSC/Mg-21	WSC/Mg-11	WSC/Mg-12	WSC/Mg-13
C−C/C=C	55.5	57.1	61.7	57.2	47.9
C−O	8.5	8.95	9.27	8.79	7.20
C=O	4.33	4.34	6.14	5.98	4.04
O=C−O	6.33	9.96	3.63	4.62	6.91

**Table 3:** Quantitative analysis of O species in WSC, WSC/Mg-21, WSC/Mg-11, WSC/Mg-12, and WSC/Mg-13 through O1s XPS spectra.

Attribution	Relative Content (%)				
	WSC	WSC/Mg-21	WSC/Mg-11	WSC/Mg-12	WSC/Mg-13
C=O	10.2	-	-	-	-
C−O	7.8	8.9	8.01	5.05	16.4
O−C=O	4.36	10.7	10.3	17.6	13.8

**Table 4:** Kinetic parameters for adsorption onto WSC/Mg-12.

Kinetic Model	Parameter	2,4-DCP
Pseudo-first order	Q_e(exp)_ (mg/g)	298.5
	k_1_ (min^−1^)	−0.0023
	Q_e(cal)_ (mg/g)	268.1
	R^2^	0.7898
Pseudo-second order	k_2_ (g·mg^−1^·min^−1^)	0.0034
	Q_e(cal)_ (mg/g)	296.7
	R^2^	0.9986
Intra-particle diffusion model	k_i1_ (g·mg^−1^·min^−1/2^)	8.877
	C_1_	142.9
	R_1_^2^	0.8872
	k_i2_ (g·mg^−1^·min^−1/2^)	1.401
	C_2_	242.6
	R_2_^2^	0.7464

**Table 5:** Constants for the Langmuir, Freundlich, Temkin, and Sips isotherms of 2,4-DCP on 2,4-DCP.

Isotherm Model	Parameter	2,4-DCP
Langmuir	Q_max_ (mg/g)	610.6
	K_L_ (L/mg)	1.225
	R^2^	0.9941
	R_L_	0.0021–0.0161
Freundlich	K_F_ (mg^(1−1/n)^ L^1/n^g^−1^)	10.29
	1/n	0.6835
	R^2^	0.9957
Temkin	B (kJ/mol)	103.1
	K_T_ (mg/L)	0.1140
	R^2^	0.9570
Sips	Q_ms_ (mg/g)	1006
	K_s_ (L/mg)^m^	0.0019
	m_s_ (g/L)	0.7721
	R^2^	0.9957

**Table 6:** Comparison of sorbents.

Adsorbent	Adsorption Capacity (mg/g)	pH Range	BET (m^2^/g)	Ref.
NC-1000@PAA	240	3–9	1097	[15]
Biomass template/SnO_2_ nanocomposite	46.0	6	19.887	[58]
ODTMA Bent/A4/1	392	2–6	-	[59]
GO-COOH@	117	4–7	4.383	[60]
LDOs	566.08	6–8	176	[56]
MBC	85.13	5–6	221.352	[57]
WSC/Mg-12	610.6	3–9	696.1	This work

**Table 7:** Adsorption experiment of coking wastewater.

Performance Indicators	Before Adsorption (mg/g)	After Adsorption (mg/g)	Removal Rate (%)
COD	2260	1819	19.5
NH_3_-N	447.5	378.2	15.5
Color intensity	-	-	80

## Data Availability

Data are available on request.

## Supplementary Materials

The following supporting information can be downloaded at: https://www.mdpi.com/article/10.3390/ijms252111761/s1.
