# Supplementary material for: Magnesium Bicarbonate–Walnut Shell Dual-Template Synthesis of Multifunctional Layered Porous Carbon for Enhanced Adsorption of Aqueous Chlorinated Organic Compounds
Source: Int J Mol Sci. 2024 Nov 1;25(21):11761. doi: 10.3390/ijms252111761 (PMC11547149; doi:10.3390/ijms252111761)
Supplement: Supplementary file 1 [file ijms-25-11761-s001.zip › ijms-3294326-supplementary.pdf]

Supporting Information

Magnesium Bicarbonate–Walnut Shell Dual-Template Synthesis of Multifunctional Layered Porous Carbon for Enhanced Adsorption of Aqueous Chlorinated Organic Compounds

Juanxue Kang[1], Xiaoli Bai[1], Junyang Leng[1], Yaxuan Lou[1,2], Daomei Chen[1], Liang Jiang[1,3], Jiaqiang Wang[1*]

1 School of Chemical Sciences & Technology, School of Materials and Energy, Institute of Frontier Technologies in Water Treatment, National Center for International Research on Photoelectric and Energy Materials, Yunnan University, Kunming 650091, China.

2 Institute of international rivers and eco-security, Yunnan University, Kunming 650091, China.

3 School of Engineering, Yunnan University, Kunming 650091, China.

* Corresponding author. Tel.: +86-871-65031567 Fax: +86-871-65031567

E-mail address: jqwang@ynu.edu.cn

Text S1. Adsorption isotherms：

The characteristics of the Langmuir isotherm can be evaluated by the equilibrium parameter or the separation parameter (R_L), which is defined by the following equation:

$$R_L=\frac{1}{1+C_iK_L} \quad \text{(S1)}$$

where Ci (mg/L) is the concentration of initial 2,4-DCP. Three types of adsorptions can be classified according to R_L values: irreversible ($R_L = 0$), favorable ($0 < R_L < 1$), linear ($R_L = 0$), or unfavorable ($R_L > 1$) [1]. In the present study R_L for 2,4-DCP remained between 0 and 1, which indicates good adsorption.

Adsorbent-adsorbate interactions is expressly taken into account by the Temkin isotherm. This model implies that all molecules' heat of adsorption might drop linearly rather than logarithmically, ignoring both extremely low and large concentration values [2]. The experimental results of Temkin models are listed in Fig. 9b and Table 6. The linearized form of the Temkin isotherm model can be written as follows:

$$Q_e=B\ln(K_TC_e) \quad \text{(S2)}$$

Where B (kJ/mol) denotes the Temkin constant of the adsorption heat, whereas K_T (mg/L) is the constant of the Temkin isotherm.

$$Q_e=\frac{Q_{ms}K_sC_e^{m_s}}{1+K_sC_e^{m_s}} \quad \text{(S3)}$$

where Q_{ms} the Sips maximum adsorption capacity (mg/g), K_S the Sips equilibrium constant $(L/mg)^m$, and m_S is the Sips model exponent.

Text S2. Materials: The walnut shell used in the present work was received from DaLi (Yunnan province, PR, China). Magnesium Hydrogen (MH) (purity: 98.0%) and Hydrochloric acid (analytical purity) were purchased from Rionlon BoHua Pharmaceutical & Chemical Co., Ltd. (Tianjin, China), Anhydrous ethanol (analytical purity) was purchased from Hengxing Chemical Reagent Manufacturing Co., Ltd. (Tianjin, China). All the other chemical reagents utilized in this study were of analytical grade and used without further purification.

Text S3. Information on instrumental parameters: The morphology of samples was

obtained by using field-emission scanning electron microscopy (FE-SEM) on an FEI Nova Nano SEM 450 microscope with accelerating voltage 10 kV (Detector: CBS, Spot: 2.5, Working distance: 5 mm). Surface element valence and chemical composition were measured by X-ray photoelectron spectroscopy (XPS) on a Thermo Fisher Scientific K-Alpha+ analyzer with mono Al Kα (hν=1486.6 eV) (vacuum level:～2 × 10-7 mba). The high resolution XPS scans were done at PE (pass energy) =30 eV (the step size: 0.1 eV). Brunauer-Emmett-Teller (BET) surface areas and pore structure data of the samples were obtained by Micromeritics TristarII3020 Surface Area and Porosity Analyzer. The light absorption properties of the samples were obtained by the UV–visible diffuse reflectance spectra (UV–vis DRS) on a Shimadzu UV- 2600 photometer.

Text S4. The TG-DTG curve demonstrates a weight loss rate of 1.4% for WSC/Mg-12 in the temperature range of 25℃ to 105℃, as depicted in Figure S5. During this stage, the mass reduction primarily arises from the evaporation of a small quantity of water present in walnut shell and magnesium bicarbonate. Furthermore, there is an observed weight loss rate of 26.03% from 105℃ to 210℃, which can be attributed to the pyrolysis of cellulose and hemicellulose in walnut shell, resulting in the liberation of volatile fractions such as CO_2 and water vapor. Additionally, within the temperature range from 210℃ to 535℃ with a weight loss rate of 11.47%, lignin undergoes cracking within the walnut shell matrix. Subsequently, between temperatures ranging from 535℃ to 800℃ with a weight loss rate of approximately 3.87%, decomposition occurs where magnesium carbonate transforms into magnesium oxide. Finally, it should be noted that WSC/Mg-12 exhibits an approximate carbon residue rateof57 .19%.

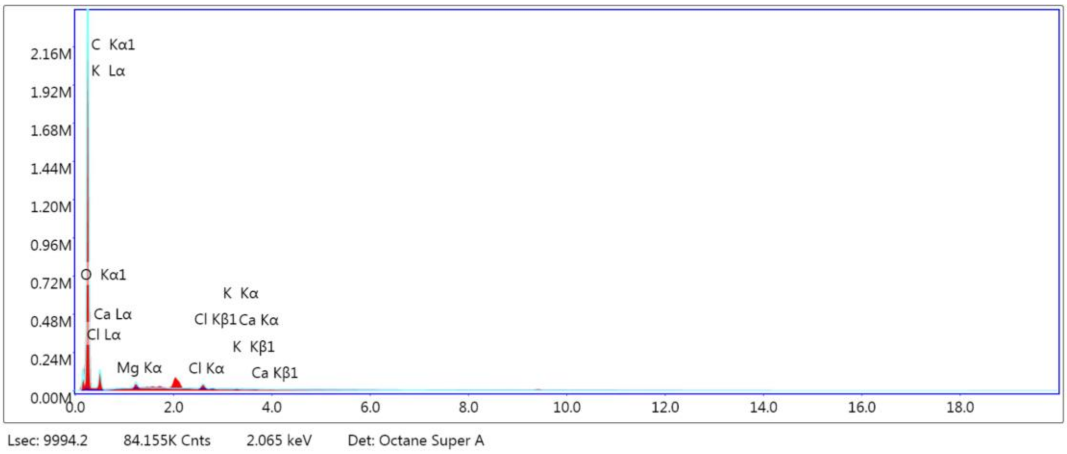

Fig. S1. The EDS pattern of WSC/Mg-12

Table S1 The EDS parameter of WSC/Mg-12

eZAF Smart Quant Results

Element	Weight%	Atomic%	Net Int.	Error%	Kratio	Z	A	F
C K	88.23	91.38	2832.50	3.36	0.6763	1.0065	0.7616	1.0000
O K	10.30	8.00	116.70	12.45	0.0112	0.9612	0.1137	1.0000
MgK	0.10	0.03	7.50	5.10	0.0009	0.8064	1.0410	1.0433
ClK	0.07	0.02	4.40	6.39	0.0006	0.8210	1.0420	1.0567
K K	0.63	0.32	54.90	4.61	0.0038	0.8858	0.6687	1.0026
CaK	0.67	0.24	59.80	1.83	0.0057	0.8107	1.0306	1.0222

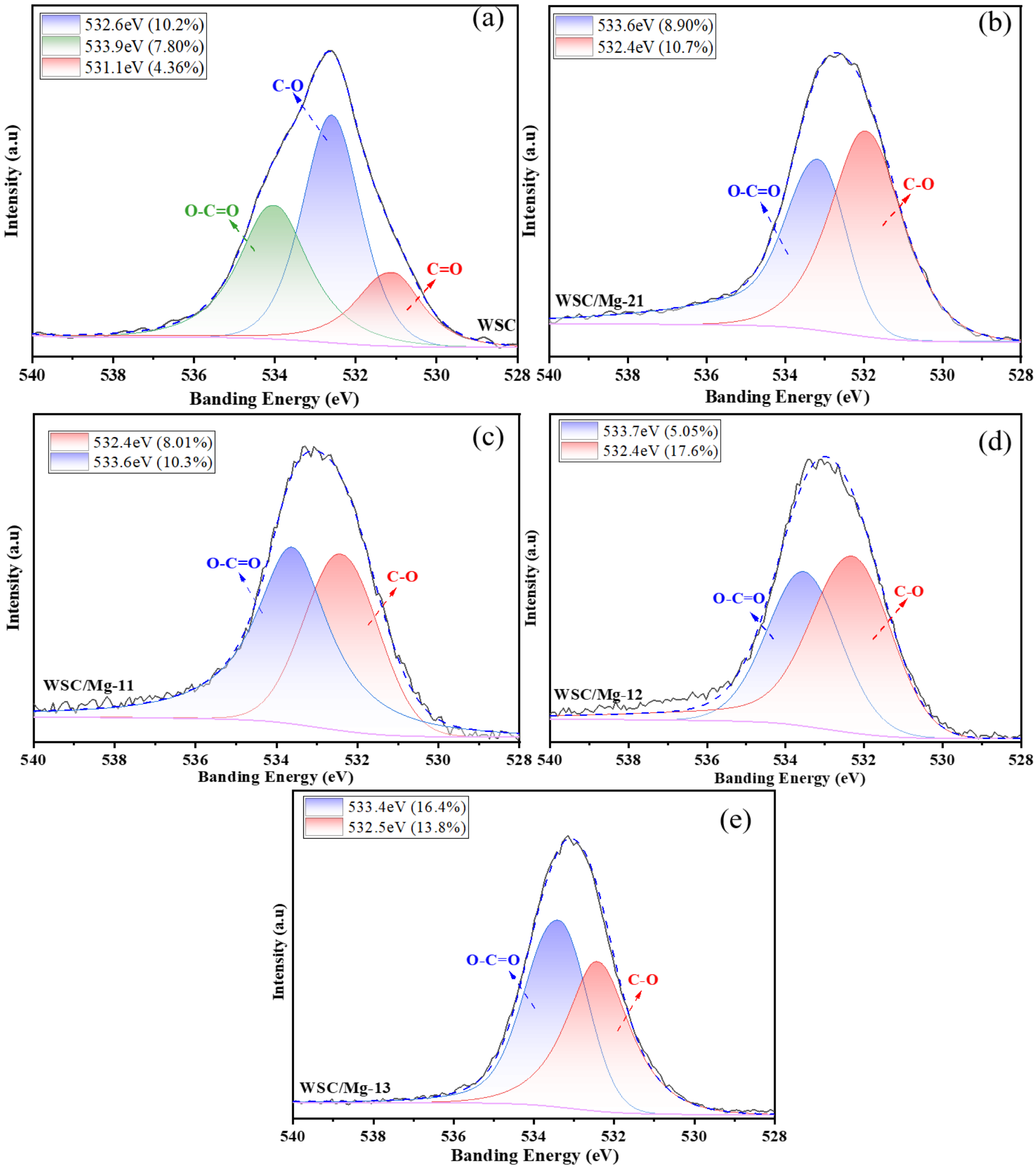

Fig. S2. (a) O1s of WSC (b) O1s of WSC/Mg-21 (c) O1s of WSC/Mg-11 (d) O1s of WSC/Mg-12 (e) O1s of WSC/Mg-13

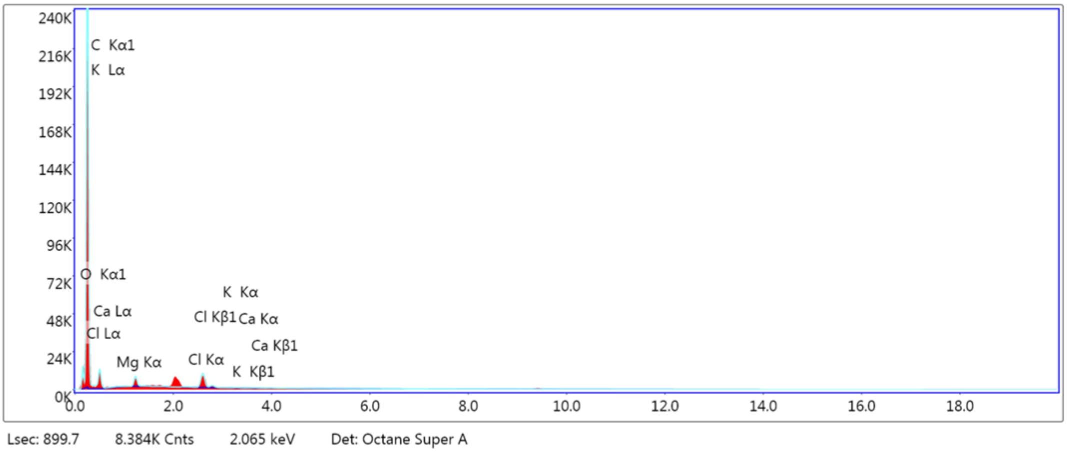

Fig. S3. The EDS patterns of WSC/Mg-12(used)

Table S2 The EDS parameter of WSC/Mg-12(used)

eZAF Smart Quant Results

Element	Weight%	Atomic%	Net Int.	Error%	Kratio	Z	A	F
C K	86.88	93.13	262.40	8.53	0.3212	1.0160	0.3638	1.0000
O K	4.79	3.86	9.90	21.22	0.0049	0.9707	0.1057	1.0000
MgK	0.09	0.05	1.50	63.63	0.0005	0.8951	0.6818	1.0038
Cl K	7.55	2.74	129.80	3.07	0.0647	0.8196	1.0337	1.0111
K K	0.30	0.10	4.10	48.34	0.0025	0.8154	1.0054	1.0218
CaK	0.39	0.13	4.70	41.21	0.0034	0.8301	1.0146	1.0259

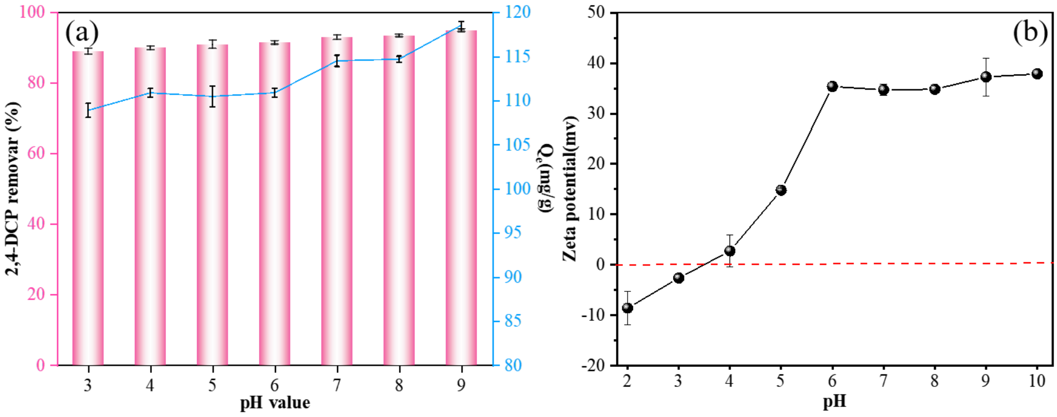

Fig. S4. (a)The effect of initial solution pH in 2,4-DCP on WSC/Mg-12; (b) Zeta potential analysis

Table S3 The physical and chemical properties of pollutant.

Name	2,4-DCP
Molecular formula	$C_6H_4Cl_2O$
Number	163.00
Absorbance value	284
CAS number	120-83-2
Chemical structure	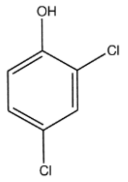

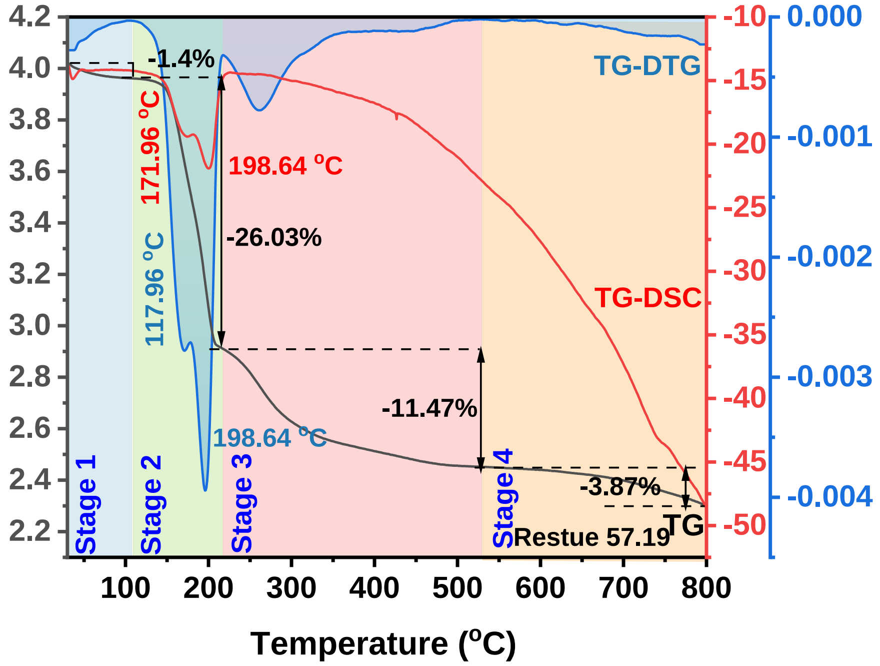

Fig. S5. The images of TG-DCS for WSC/Mg-12

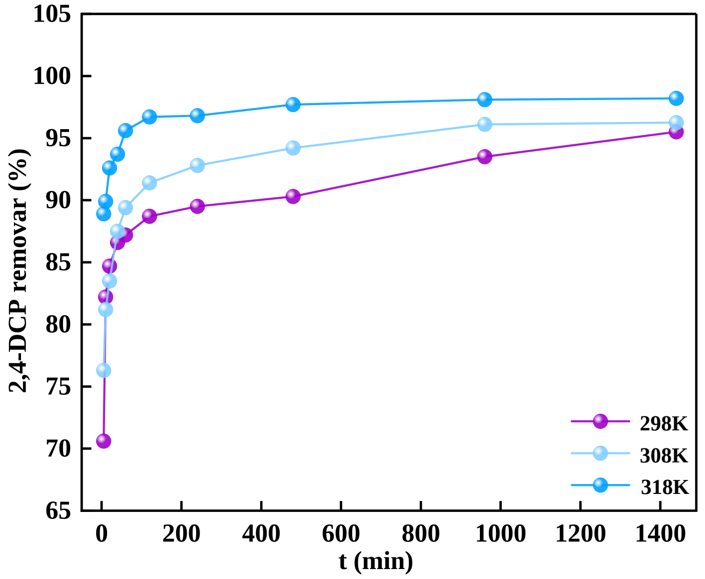

Fig. S6. Effects of different temperatures on 2,4-DCP adsorption by WSC/Mg-12

[1] Cheng, Yizhen, Anwen Li, Wei Shi, and Longshan Zhao. "Magnetic chitosan-functionalized waste carton biochar composites for efficient adsorption of anionic and cationic dyes." Chemical Engineering Journal 481 (2024): 148535. https://doi.org/10.1016/j.cej.2024.148535.
[2] Xu, Jiang, Xue Liu, Gregory Victor Lowry, Zhen Cao, Heng Zhao, John L. Zhou, and Xinhua Xu. "Dechlorination mechanism of 2, 4-dichlorophenol by magnetic MWCNTs supported Pd/Fe nanohybrids: rapid adsorption, gradual dechlorination, and desorption of phenol." ACS applied materials & interfaces 8, no. 11 (2016): 7333-7342. https://doi.org/10.1021/acsami.5b11859.
